# Activation of EphA4 and EphB2 Reverse Signaling Restores the Age-Associated Reduction of Self-Renewal, Migration, and Actin Turnover in Human Tendon Stem/Progenitor Cells

**DOI:** 10.3389/fnagi.2015.00246

**Published:** 2016-01-06

**Authors:** Cvetan Popov, Julia Kohler, Denitsa Docheva

**Affiliations:** Experimental Surgery and Regenerative Medicine, Department of Surgery, Ludwig Maximilians UniversityMunich, Germany

**Keywords:** tendon aging and degeneration, TSPC, ephrins, cell migration, actin dynamics

## Abstract

Tendon tissues, due to their composition and function, are prone to suffer age-related degeneration and diseases as well as to respond poorly to current repair strategies. It has been suggested that local stem cells, named tendon stem/progenitor cells (TSPCs), play essential roles in tendon maintenance and healing. Recently, we have shown that TSPC exhibit a distinct age-related phenotype involving transcriptomal shift, poor self-renewal, and elevated senescence coupled with reduced cell migration and actin dynamics. Here, we report for the first time the significant downregulation of the ephrin receptors EphA4, EphB2 and B4 and ligands EFNB1 in aged-TSPC (A-TSPC). Rescue experiments, by delivery of target-specific clustered proteins, revealed that activation of EphA4- or EphB2-dependent reverse signaling could restore the migratory ability and normalize the actin turnover of A-TSPC. However, only EphA4-Fc stimulation improved A-TSPC cell proliferation to levels comparable to young-TSPC (Y-TSPC). Hence, our novel data suggests that decreased expression of ephrin receptors during tendon aging and degeneration limits the establishment of appropriate cell-cell interactions between TSPC and significantly diminished their proliferation, motility, and actin turnover. Taken together, we could propose that this mechanism might be contributing to the inferior and delayed tendon healing common for aged individuals.

## Introduction

Tendon injuries due to excessive mechanical stress or tissue aging and/or degeneration are common and present a significant challenge for orthopedic surgery. Aged and/or degenerated tendons respond poorly to classical medicinal treatments, which often leads to rupture reoccurrence. Until now, several major factors contributing, directly, or indirectly, to tendon aging and degeneration were identified: disturbance of extracellular matrix turnover; decreasing cell numbers and metabolic activity; tenocyte dedifferentiation; and depletion or senescence of the local stem/progenitor cell pool (Jozsa and Kannus, 1997; Kaeding and Best, 2009; Zhou et al., 2010; Kohler et al., 2013).

Tendon stem/progenitor cells (TSPCs) were first reported in (Bi et al., 2007) as plastic adherent cells that possess strong clonogenic potential and express classical stem cell markers, while maintaining the expression of typical tendon-lineage genes, such as Scleraxis and tenomodulin (Bi et al., 2007; Kohler et al., 2013). Additionally, these cells are able to differentiate to three different lineages *in vitro* and more importantly, can form tendon-like tissue *in vivo* (Bi et al., 2007). In the same year, by using chimeric tendon-GFP rat models, (Kajikawa et al., 2007) proposed that tendon healing is carried out mainly by such local tendon progenitor cells, which actively migrate to the wound site and engage in cell proliferation. However, others and we have found that TSPC features alter during tendon aging and degeneration (Zhou et al., 2010; Kohler et al., 2013). Aged TSPC (A-TSPC) display a profound self-renewal deficit accompanied with premature entry into senescence and substantial changes in their transcriptome, especially in genes regulating cell adhesion, migration and cytoskeleton, but unaltered multipotential (Kohler et al., 2013). Furthermore these cells exhibit severely dysregulated cell–matrix interactions, motility and actin dynamics (Kohler et al., 2013).

In recent years, the role of ephrins and their signaling in regulating numerous cellular processes has been recognized in different cell types and tissues. Ephrins are receptor tyrosine kinases that mediate short-range cell-cell communication. For their activation, a binding between the membrane-bound ephrin receptor (Eph) and ephrin ligand (EFN) located on the surface of the neighboring cell is required. There are 9 EphA and 5 EphB receptors, and 5 EFNA and 3 EFNB ligands expressed in humans. Typically, EphA receptors bind to EFNA ligands and EphB receptors bind to EFNB ligands; however, EphA4 and EphB1 receptors can bind to both EFNA and EFNB ligands (Egea and Klein, 2007). Eph-EFN bond initiate simultaneously bidirectional signaling in the receptors (forward) and ligands (reverse) expressing cells that can activate key cellular kinases, such as focal adhesion kinase (FAK), extracellular signal-regulated kinases (ERK), Akt, c-Jun N-terminal kinases (JNKs) and p38 mitogen-activated protein kinases (p38), and thereon can influence cell self-renewal, migration, and actin turnover (Murai and Pasquale, 2003; Pasquale, 2010; Arthur et al., 2011).

Ephrin receptor-ligand interactions and their diverse roles have been the best studied in neuronal and cancerous cells (Genander and Frisen, 2010). Ephrins were linked to regeneration of the central neuronal system due their functions in neuronal connections and axon guidance (Du et al., 2007). Certain ephrin members have been associated with cancer cell migration and tumor progression (Guo et al., 2006).

Few studies have also elaborated on the role for ephrins and their signaling in musculoskeletal tissues. For example in bone, ephrins can positively influence osteoblast differentiation (Zhao et al., 2006; Xing et al., 2010), but suppress osteoclastogenesis by affecting TRAP, cathepsin K and integrin β3 signaling (Zhao et al., 2006; Cheng et al., 2012; Stiffel et al., 2014). In muscle development, ephrin interactions are required for the appropriate formation of neuromuscular junctions, nerve branching and topographic innervation within individual muscles, as well as for myoblast directed migration to the dorsal and ventral limb muscles (Swartz et al., 2001; Stark et al., 2011). Interestingly, it has been found that ephrins can also affect the functions of tissue-resident stem cells. Arthur et al. (2011) reported that activation of EFNB1 and EFNB2 reverse signaling inhibit the attachment and spreading of bone-marrow mesenchymal stem cells, while stimulation of EphB2 and EphB4 forward signaling promotes their migration. Goichberg et al. (2011) and Li and Johnson (2013) showed that EphA2/EFNA1 and EFNA5 can enhance the migration of human and bovine cardiac stem cells, correspondingly. In a follow up study, Goichberg et al. (2013) found that disturbed EphA2/EFNA1 signaling is related to age-associated senescence and reduced migration of human cardiac progenitor cells, and demonstrated that overexpression of EphA2 in these cells can rescue their senescent and migratory phenotype.

Despite of the broader knowledge in other tissues, up-to-date ephrin family expression and functions in tendon tissues and cells essentially remain unknown. Only one developmental paper, based on *in situ* hybridization analyses, has reported during embryonal chick development a strong EphA4 expression within the tendon core (D’Souza and Patel, 1999). Interestingly, our previous microarray data suggested differential expression levels of several ephrin members in A-TSPC in comparison to the control young-TSPC (Y-TSPC; Kohler et al., 2013).

Cumulatively, this strongly motivated us to examine for the first time the expression pattern of this neglected family in TSPC. Our main aims in this study were: (i) to characterize the ephrin expression profile of TSPC *in vitro*; (ii) to identify key candidates among the expressed ephrin members that are dysregulated in A-TSPC versus Y-TSPC; and (iii) to investigate if the selected ephrin members can contribute to restoring their aging phenotype.

## Materials and Methods

### Cell Isolation and Culture

The isolation and complete characterization of human Y- and A-TSPC was reported in Kohler et al. (2013, under Ethical grant No. 166-08 LMU Medical Faculty). In brief, TSPC were obtained from non-ruptured Achilles tendons from four young and 12 elder human donors as tendon tissue biopsies were minced into small pieces, enzymatically digested with 0.15% collagenase II (Worthington, USA) in culture medium at 37°C overnight, filtered with sterile nylon mesh (100 μm pore size) and centrifuged at 500 *g* for 10 min. Then, cell pellets were suspended and expanded in DMEM/Ham’s F-12 (1:1 mixture) supplemented with stabile glutamine, 1× MEM amino acids (all from Merck Millipore, USA), 10% FBS, and 1% L-ascorbic acid-2-phosphate (both from Sigma–Aldrich, Germany), and the TSPC were cultivated in a humidified incubator at constant 37°C and 5% CO_2_. During the initial cultivation, all individual cell lines were monitored for cell yield, morphology and expansion kinetics. Since within each group, the cell lines were very comparable, equal group size of three representative donor-derived TSPC were formed in order to carry out in-depth ephrin analyses. All experiments were performed with TSPC at passages 4–6.

Activation of ephrin-dependent signaling in A-TSPC was done based on (Kaneko and Nighorn, 2003; Arthur et al., 2011) protocol with minor modifications. First, 1 μg/ml recombinant human ephrin-Fc chimera (EphA4, EphB2, EphB4, and EFNB1-Fc, all R&D systems, USA) or control-Fc proteins were clustered with polyclonal anti-human Fc antibody (molar ratio 5:1, Dianova, Germany) in complete culture media at room temperature for 30 min. Then, A-TSPC were harvested from tissue culture dishes, counted, resuspended, and incubated in ephrin-Fc or Fc-control media for 30 min in humidified incubator. Thereafter, cells were plated for various experiments and the treated A-TSPC were supplemented every second day with fresh media, containing 1 μg/ml clustered ephrin-Fc and Fc-control. In each experiment, Y-TSPC at the same passage number was used as positive control.

### Quantitative PCR Analysis

RNA isolation and cDNA synthesis were performed similar to (Popov et al., 2011): RNA was extracted with RNeasy Mini Kit (Qiagen, Gemany) and 1 μg total RNA was used for cDNA synthesis with AMV First-Strand cDNA Synthesis Kit following the manufacturer’s instructions (Thermo scientific, USA). Quantitative PCR analysis for the complete ephrin family expression in human Y- and A-TSPC was done with RealTime Ready Custom panel array (Roche, Germany). Each array consisted of 9 EphA (1–8 and 10), 5 EphB (1–4 and 6), 5 EFNA (1–5), and 3 EFNB (1–3) genes and three housekeeping genes GAPDH, HPRT, and SDHA. For calculation of the relative gene expression, ratio between targeted gene and GAPDH was made. Data consisted of three independent repeats with all three Y- and A-TSPC donors (*n* = 9).

### Cytochemistry

Y- and A-TSPC plated and cultured on 20 μg/ml collagen 1-coated glass slides (BD Bioscience, USA) for 48 h were fixed with 4% paraformaldehyde (Merck, Germany) or 7% formalin/PHEM (6 mM PIPES, 25 mM HEPES, 10 mM EGTA, 3 mM MgCl2, pH 6.1, all Sigma–Aldrich) solutions. Then, cells were permeabilized with 0.1% Triton X100 (Sigma–Aldrich) and blocked with 3% BSA (Millipore, USA). Primary antibodies against EphA4 (Abnova, Germany), EphB2 (Biomol, Germany), EphB4 (Thermo scientific), EFNB1 and EFNB2 (both Sigma–Aldrich) were applied overnight at 4°C. Next, secondary Alexa Flour 488-conjugated antibodies and DAPI were used (all Life technologies). For negative control, cell-seeded slides were incubated only with the secondary antibody and DAPI. For F-actin staining, formalin/PHEM fixed cells were incubated with phalloidin-AF546 (Life technologies) for 40 min at room temperature. Photomicrographs were taken with Axiocam MRm camera on AxiovertS100 microscope (Carl Zeiss, Germany). Staining experiments were repeated two independent times for all three Y- and A-TSPC donors (*n* = 6).

### Western Blot Analysis

Total protein from Y- and A-TSPC (clustered EphA4 and EphB2, and Fc-control) was isolated with RIPA-buffer (0.1% SDS, 1% Na-DOC, 1% Triton X-100, 50mM Tris-HCl pH8.2, 150mM NaCl, 10mM EDTA, 20mM NaF, 1mM Na_3_VO_4_) supplemented with complete protease inhibitors (Roche). Proteins (20 μg) were separated on SDS gels, transferred onto PVDF membrane and blocked with 5% skim milk (Merck) for 1 h at room temperature. Primary antibodies against human EphA4 (Abnova), EphB2 (Biomol), EphB4 (Thermo scientific), EFNB1 and EFNB2 (both Sigma–Aldrich); phospho-FAK (Thermo scientific), total FAK, total and phospho-ERK1/2; total and phospho-Akt; total and phospho-p38; total and phospho-Jnk (all Cell Signaling, USA), and GAPDH (Merck) were applied overnight at 4°C. Then, membranes were incubated with corresponding secondary HRP-conjugated antibodies (Cell Signaling) for 1 h at room temperature and consequently with ECL solution (GE Healthcare, USA). Photomicrographs were taken on ImageQuant LAS 4000 mini (GE Healthcare) as band intensities were quantified with ImageProPlus4 software program (Media Cybernetics, USA). Western blot experiments were preformed two independent times with all three Y- and A-TSPC donors (*n* = 6).

### Self-Renewal Analysis

Tendon stem/progenitor cell self-renewal was assessed with WST-1 proliferation and colony forming unit (CFU) assays as described in (Kohler et al., 2013). For cell proliferation, 3 × 10^3^ cells/ cm^2^ of Y- and A-TSPC (EphA4-Fc, EphB2-Fc, and Fc-control) were plated in complete culture medium containing 0.2% FBS, with or without clustered EphA4 and EphB2 protein for 7 days. Thereafter, WST-1 reagent (Roche) was applied for 4 h and optical density (OD) was measured at 420 and 620 nm using a Microtiter Reader (Thermo Scientific). Cell proliferation was calculated in percentage to Y-TSPC. WST-1 assay was reproduced three independent times with all Y- and A-TSPC donors, as each experiment was done in triplicates (*n* = 9). For the CFU assay, 20 cells/ cm^2^ of Y-, and A-TSPC (EphA4-Fc, EphB2-Fc, and Fc-control) were plated in 10 cm culture dishes for 12 days in complete media supplemented with or without EphA4 and EphB2 clustered protein. Then, cell colonies were visualized with 0.5% crystal violet/methanol staining and counted. CFU efficiency was estimated as percentage of counted colonies to the number of plated cells. CFU experiment was performed three independent times of all Y- and A-TSPC donors in duplicates (*n* = 9).

### Migration Analysis

Migration analysis was performed similarly to (Kohler et al., 2013) using Axiocam ICc3 camera mounted on Axiovert S100 inverted microscope (Carl Zeiss) equipped with biochamber (PeCon, Germany) providing stable culture conditions. For random migration, 1.5 × 10^3^ cells/ cm^2^ of Y- and A-TSPC (EphA4-Fc, EphB2-Fc, and Fc-control) were seeded in 6-well plates and incubated for 2 h prior imaging. Time-lapse was performed with four frames per h for 2 days. The image data was extracted with AxioVisionLE software (Carl Zeiss) and individual cell tracks were analyzed with ImageJ V1.48 software. Random migration was expressed by calculation of the forward migration index [FMI; the ratio of the vector length to the migratory starting point based on (Sip et al., 2014)]. Results of random TSPC migration consist of three independent repeats with all Y- and A-TSPC donors (*n* = 9; total of 150 tracks per donor type). For the scratch assay, 1 × 10^4^ cells/ cm^2^ of Y- and A-TSPC (EphA4-Fc, EphB2-Fc, and Fc-control) were plated in 6-well dishes and let to form confluent monolayers for 2 days. Prior imaging, the layers were scratched multiple times. Time-lapse was performed with four frames per h for 3 days. The initial scratch length and time needed for gap bridging were measured and used for calculation of cell velocity. The scratch assay was reproduced three independent times for each donor group as each experiment was done in triplicates (*n* = 9, 36 scratches per donor type).

### Quantification of Cell Area and Actin Dynamics

Quantification of Y- and A-TSPC (EphA4-Fc, EphB2-Fc, and Fc-control) cell area was obtained from F-actin images by measuring 50 cells from each donor with the polygonal tool of the ImageProPlus4 software (Media Cybernetics). Actin dynamics of Y- and A-TSPC (EphA4-Fc, EphB2-Fc, and Fc-control) was analyzed by latrunculin A experiment, carried out as described in (Kohler et al., 2013). Latrunculin A inhibits actin polymerization by sequestering monomeric G-actin and thereby disrupts the turnover of actin filaments. In brief, the four different TSPC groups, Y- and A-TSPC with EphA4-Fc, EphB2-Fc, and Fc-control, (5.5 × 10^3^ cells/cm^2^) were grown in 96 well plates and pre-cultured for 48 h. Then, cells were treated with 0.4 μM latrunculin A (Sigma–Aldrich), fixed at different time intervals (0, 2, 5, 8, 10, 20, 30, and 60 min), stained with 0.6 μM phalloidin-546 (Thermo Scientific) and fluorescence signals were recorded at 573 nm using a SAFIRE2 microplate reader (Tecan, Germany). In each group, F-actin content at time point 0 was set to 100%. Latrunculin A analyses were reproduced three independent times for all Y- and A-TSPC donors in triplicates (*n* = 9).

### Statistical Analysis

In the study 3 Y-TSPC and 3 A-TSPC representative lines were used in all experiments. The quantitative data was generated out of all three different donors per group and furthermore each donor was used in 3 (*n* = 9) or 2 (*n* = 6) independent experiments. Statistical evaluation was performed using the GraphPrism software (GraphPad, La Jolla, CA, USA). Final graphs and bar charts show mean values ± SD of two or three independent experiments for the donor group. Within each of the independent experimental repeats, the individual donors were represented in triplicates, however, the replicates were calculated as mean value for the donor. Unpaired *t*-test was used for two group analysis and Tukey’s one-way ANOVA was applied for multi group statistical testing. A *p*-value < 0.05 was considered statistically significant (^∗^*p* < 0.05; ^∗∗^*p* < 0.01, ^∗∗∗^*p* < 0.001).

## Results

### Expression Levels of Several Ephrin Members were Significantly Downregulated in A-TSPC

**FIGURE 1:**
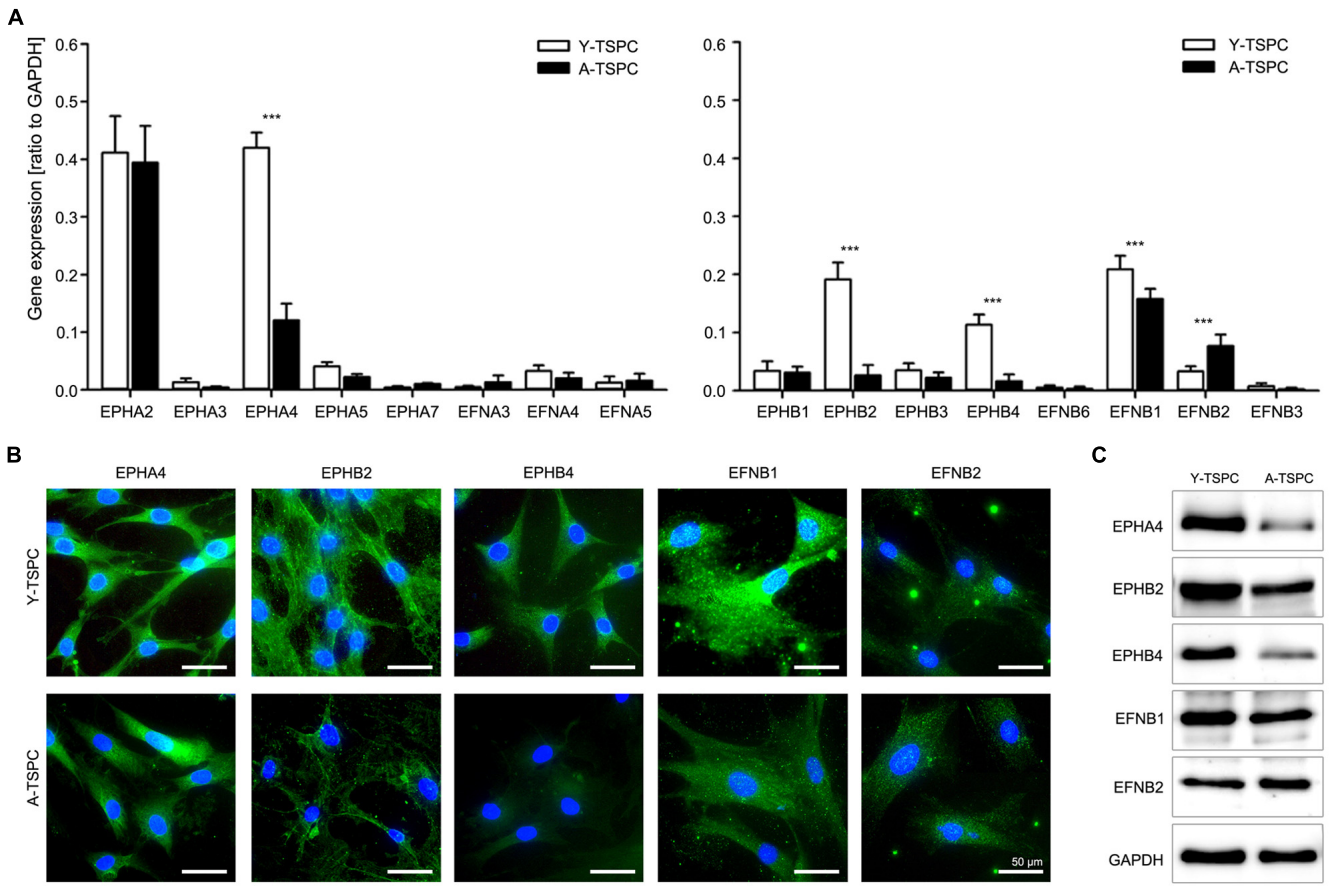
**Ephrin expression in tendon stem/progenitor cells (TSPCs). (A)** Comparison between Y- and A-TSPC basal ephrin family expression by quantitative PCR array. Data consists of three independent repeats with all three Y-and A-TSPC donors (*n* = 9, ^∗∗∗^*p* ≤ 0.001). **(B)** Cytochemical and **(C)** western blot protein analysis for EphA4, EphB2, EphB4, EFNB1, and EFNB2 in Y- and A-TSPC (*n* = 6; full blot shown in Supplementary Figure S1). Representative images; bars = 50 μm.

We first compared the whole ephrin family expression profile in Y- and A-TSPC by using quantitative PCR (**Figure 1A**). Our results demonstrated that in comparison to Y-TSPC, A-TSPC expressed significantly lower levels of EphA4 (3.5 folds), EphB2 (7 folds), and EphB4 (7 folds) receptors, and EFNB1 (1.3 folds) ligand, but increased levels of EFNB2 (2.3 folds). These expression changes were further verified on protein level by immunocytochemical staining (**Figure 1B**) and western blotting (**Figure 1C**), which confirmed the downregulation of EphA4, EphB2, EphB4, and EFNB1, and upregulation of EFNB2 expression in A-TSPC. Taken together, our novel data evidently showed that during tendon aging and degeneration, EphA4, EphB2, EphB4, and EFNB1 mRNA and protein expression levels are significantly downregulated in TSPC.

### Analysis of Activation of Key Cellular Kinases in A-TSPC Selects EphA4 and EphB2 as Main Candidates of Interest

**FIGURE 2:**
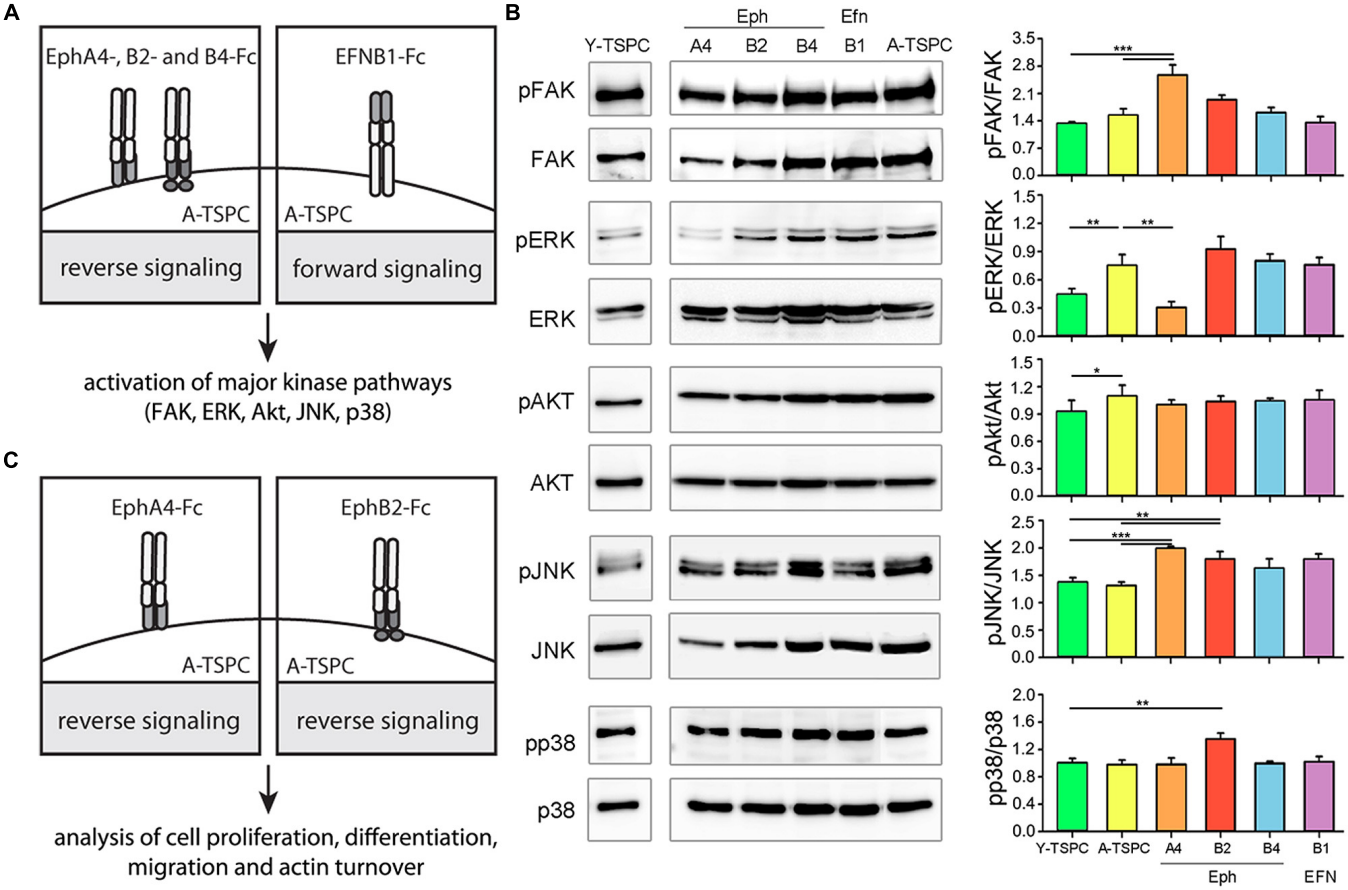
**Experimental design and western blot analysis of key cellular kinases in TSPC. (A)** Schematic presentation of A-TSPC stimulation with clustered ephrins. **(B)** Western blot analysis of phosphorylated and total FAK, ERK, Akt, JNK, and p38 kinases in Y-TSPC and EphA4-Fc, EphB2-Fc, EphB4-Fc, EFNB1-Fc, and control-Fc A-TSPC. Data consists of two independent repeats with all three Y-and A-TSPC donors (*n* = 6, ^∗^*p* < 0.05; ^∗∗^*p* < 0.01; ^∗∗∗^*p* < 0.001). **(C)** Experimental study design.

In order to investigate the importance of EphA4, EphB2, EphB4, and EFNB1 downregulation for TSPC aging and degeneration, we next designed rescue experiments based using established protocols (refer to Materials and Methods). We first stimulated externally A-TSPC with EphA4, EphB2, EphB4, and EFNB1 clustered proteins and then analyzed the activation of their downstream signaling in the cells (**Figure 2A**). As mentioned in the introduction, the main downstream effectors of ephrin-mediated signaling are the cellular kinases FAK, ERK, Akt, JNK, and p38 (**Figure 2B** and Supplementary Figure S1). Direct comparison in-between basal kinase activities demonstrated that ERK and Akt phosphorylation was significantly elevated in A-TSPC. EphA4-Fc stimulation of A-TSPC led to increased FAK and JNK activity and more importantly, to reduced ERK phosphorylation to levels comparable to Y-TSPC. Stimulation with EphB2-Fc resulted in significantly increased phosphorylation levels of JNK and p38 kinases in A-TSPC. These results suggests that EphA4 and EphB2 signaling overlaps mainly in activation of JNK kinase; however, these both ephrins demonstrated clear difference in ERK, FAK, and p38 activation. In comparison to EphA4 and EphB2, activation with EphB4-Fc and EFNB1-Fc did not show robust effect on A-TSPC kinase activity. Therefore, in our next experiments, we continued only with EphA4-Fc and EphB2-Fc, and investigated their effect on A-TSPC self-renewal, migration and actin dynamics (refer to **Figure 2C**).

### EphA4-Fc Signaling, but not EphB2-Fc, Positively Affects A-TSPC Self-Renewal

**FIGURE 3:**
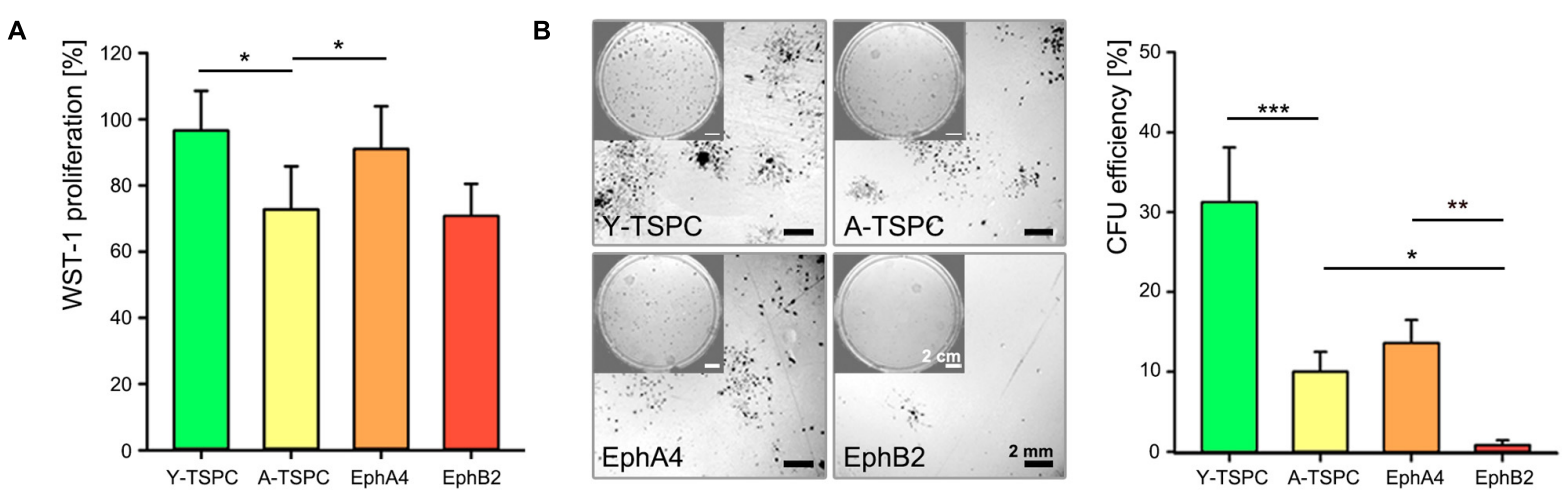
**Self-renewal potential of EphA4-Fc and EphB2-Fc A-TSPC. (A)** WST-1 proliferation and **(B)** CFU analysis of Y-TSCP, EphA4-Fc, EphB2-Fc, and control-Fc A-TSPC. Data consists of three independent repeats with all three Y-and A-TSPC donors (*n* = 9, ^∗^*p* < 0.05; ^∗∗^*p* < 0.01; ^∗∗∗^*p* < 0.001).

We investigated the effect of EphA4-Fc and EphB2-Fc on TSPC self-renewability by performing short-term cell proliferation (WST-1; **Figure 3A**) and colony-forming units (CFU) assays (**Figure 3B**). A-TSPC exhibited slower proliferation rate and clonogenic ability. Interestingly, when treated with clustered EphA4-Fc A-TSPC proliferation significantly increased to levels comparable to Y-TSPC, while supplementation with EphB2-Fc had no effect. The addition of EphA4-Fc to A-TSPC had tendency to elevate their CFU numbers; however, it did not lead to significant change (*p* = 0.06). In comparison to EphA4-Fc, the second candidate EphB2-Fc showed a significant difference as treatment with this ephrin inhibited the A-TSPC clonogenic potential with approximately 12-folds, thus suggesting that EphA4- and EphB2-mediated reverse signaling cascades diverge in their cellular functions.

### EphA4-Fc and EphB2-Fc Restore the Migration Deficit of A-TSPC

**FIGURE 4:**
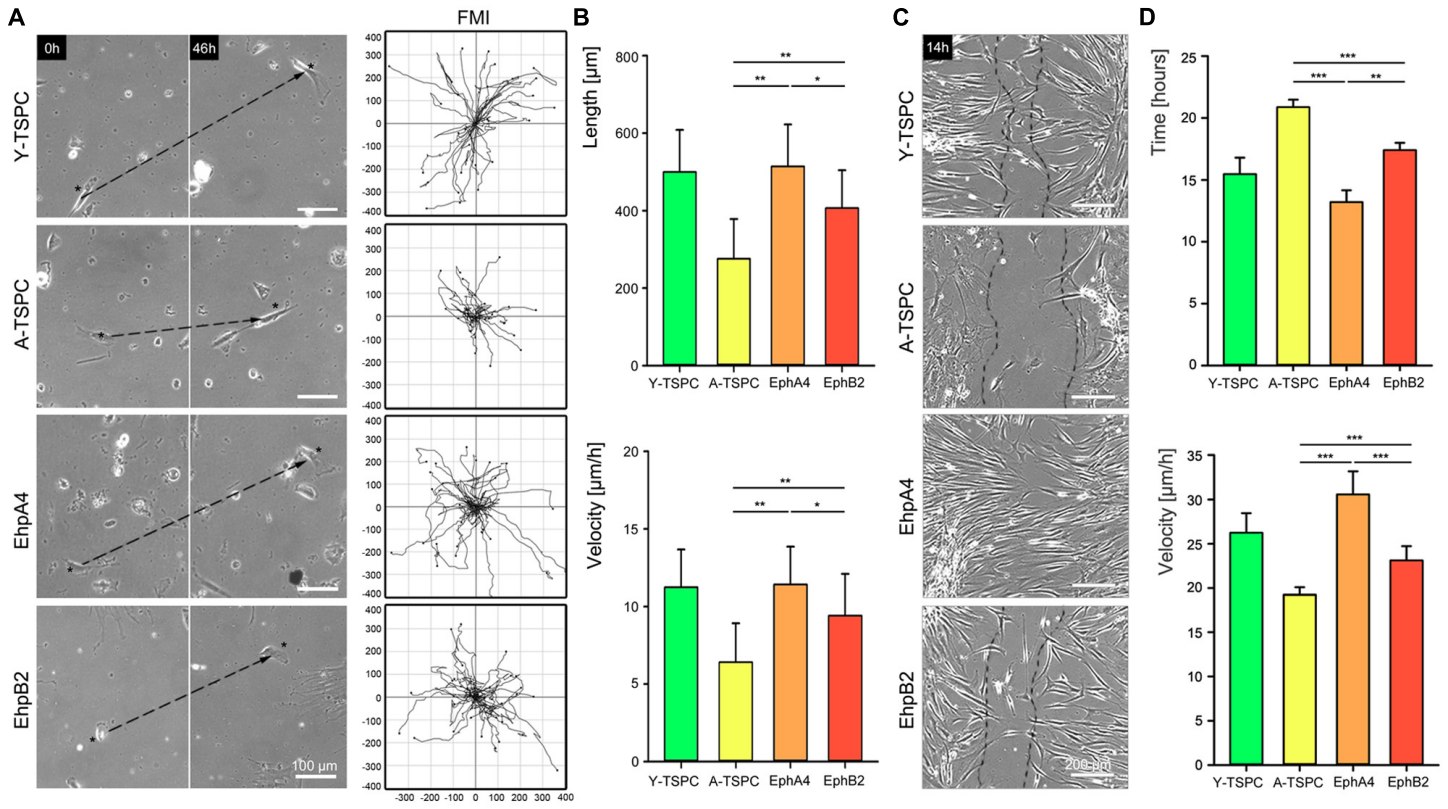
**Migration of EphA4-Fc and EphB2-Fc A-TSPC. (A)** Representative images of random migration (dash lines show ecludian distance) and calculation of forward migration index (FMI). Bars = 100 μm. **(B)** Quantification of random migration (distance and velocity) (*n* = 9, total of 150 tracks per donor type). **(C)** Representative scratch assay of Y-TSCP, EphA4-Fc, EphB2-Fc, and control-Fc A-TSPC. Bars = 200 μm. **(D)** Estimation of scratch bridging time and cell velocity (*n* = 9, total of 36 scratches per donor type, ^∗^*p* < 0.05; ^∗∗^*p* < 0.01, ^∗∗∗^*p* < 0.001).

In order to determine whether EphA4-Fc and EphB4-Fc have positive influence, we next performed random migration and scratch assays (**Figure 4**). The random migration, represented by FMI confirmed that A-TSCP are indeed less migratory than Y-TSPC and showed that the addition of both EphA4-Fc and EphB2-Fc enhances their cell motility (**Figure 4A**). Moreover, quantification of accumulated length and velocity clearly indicated that the observed effect is significant (**Figure 4B**). However, in-between both types of receptors, EphA4 was more potent inducer of TSPC migration. This finding was further validated by scratch assay (**Figures 4C,D**) revealing that EphA4-Fc A-TSPC closed first the distance between the two fronts (13.2 ± 0.9 h), followed by Y-TSPC (15.4 ± 1.3 h), EphB2-Fc A-TSPC (17.4 ± 0.5 h), and last A-TSPC (20.9 ± 0.6 h) (**Figure 4C**). In summary, we can report for the first time that activation of EphA4 or EphB2-dependent reverse signaling can rescue the migration deficit of A-TSPC, furthermore, EphA4-Fc stimulated A-TSPC performed even better than Y-TSPC.

### EphA4-Fc and EphB2-Fc Improve A-TSPC Actin Dynamics

**FIGURE 5:**
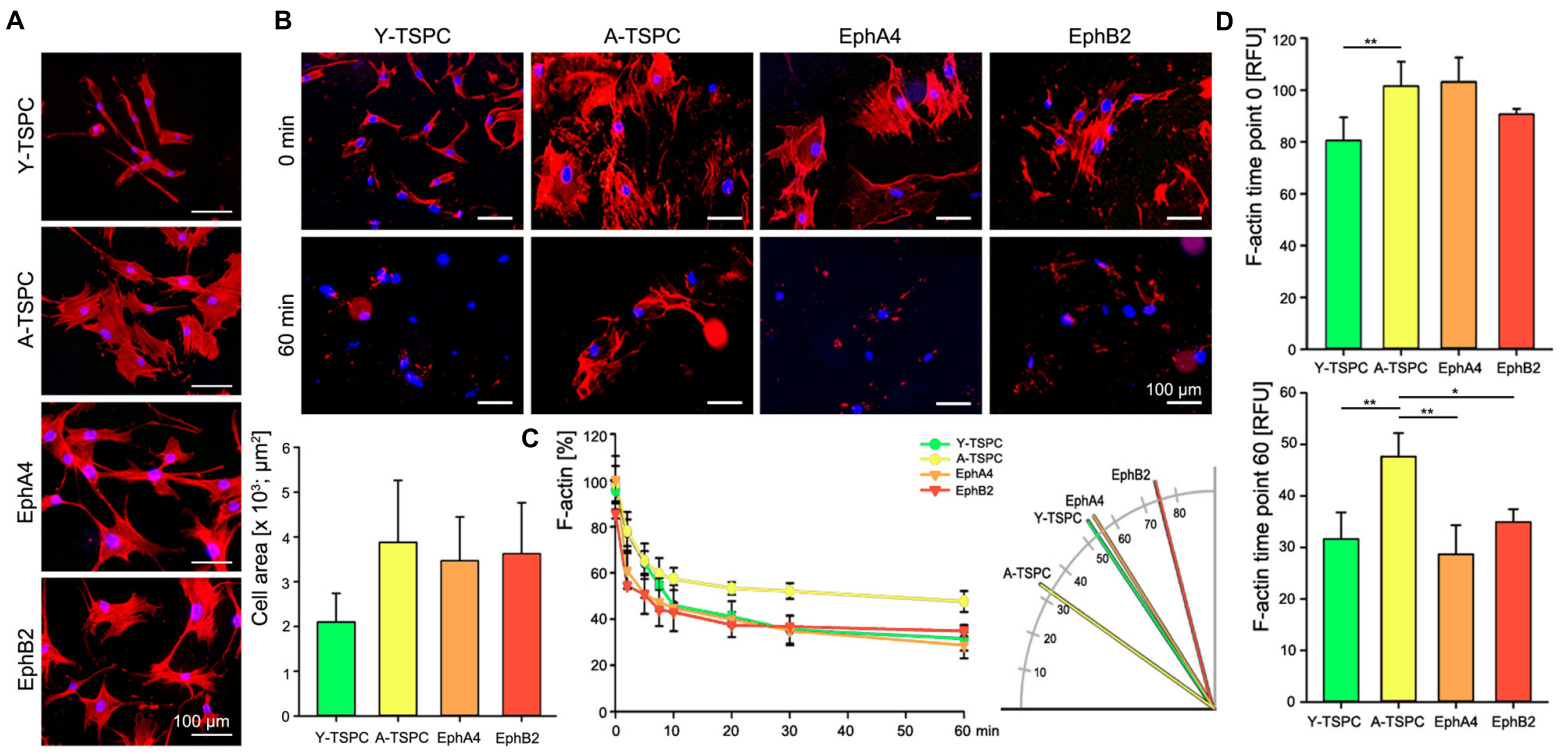
**Actin dynamics of EphA4-Fc and EphB2-Fc A-TSPC. (A)** Representative images of F-actin staining and quantification of the cell area of Y-TSPC, EphA4-Fc, EphB2-Fc, and control-Fc A-TSPC (*n* = 9, total of 50 cells per donor). **(B)** Representative images of Y-TSPC, EphA4-Fc, EphB2-Fc, and control-Fc A-TSPC at seven time points during latrunculin A treatment. **(C)** Quantification of actin filament dynamics in a time-dependent manner and curve declination angle. **(D)** Total actin content at the beginning (0 min) and at the end (60 min) of the latrunculin A experiment (*n* = 9, ^∗^*p* < 0.05; ^∗∗^*p* < 0.01).

Finally, we performed phalloidin staining for F-actin and compared the actin filament dynamics in the four TSPC groups by treating them with latrunculin A in a time-dependent manner. First, we investigated changes in cell area and we found neither EphA4-Fc nor EphB2-Fc affected the cell area habituated by A-TSPC (**Figure 5A**). In contrast, our latrunculin a analyses, performed 48 h after A-TSPC treatment with the corresponding ephrins, indicated reduced F-actin content visualized by phalloidin staining at 60 min (**Figure 5B**). Quantification of time-dependent F-actin decrease and curve declination angle (**Figure 5C**) showed that both EphA4-Fc and EphB2-Fc significantly improved the actin turnover of A-TSPC to levels comparable to Y-TSPC. **Figure 5D** represents the basal levels of F-actin in the four different groups prior the latrunculin A treatment (upper graph, time point 0 min). However, the EphA4-Fc- or EphB2-Fc-treated A-TSPC, when challenged with LatA, were able to normalize their F-actin content to levels similar to Y-TSPC (lower graph, time point 60 min).

In summary, our novel findings demonstrate that activation of EphA4- and EphB2-dependent reverse signaling augments the motility and actin turnover of A-TSPC, but only EphA4 rescues their cell proliferation deficit.

## Discussion

In the current study, we report for the first time that A-TSCP have dysregulated cell–cell interactions mediated by the ephrin family. By comparing Y- to A-TPSC we found that the expression of several ephrin members is significantly changed. Next, by carefully examining the role of two main candidates, namely the receptors EphA4 and EphB2, we could demonstrate that by activating their reverse signaling, via external delivery of the clustered receptors, we can normalize several of the A-TSCP deficits.

The importance of ephrin receptor-ligand interactions has increasingly been recognized not only in the neuronal system (Du et al., 2007) and cancer (Guo et al., 2006), but also in musculoskeletal tissues such as bone (Zhao et al., 2006; Cheng et al., 2012; Stiffel et al., 2014) and muscle (Swartz et al., 2001; Stark et al., 2011). As mentioned in the introduction, only one paper so far reported on the expression of EphA4 during tendon morphogenesis in chick limbs (D’Souza and Patel, 1999). Recent study focusing on periodontal ligament fibroblasts and osteoblasts showed important strain-dependent involvement of EphB4/EFNB2 in modulating osteogenesis during tooth movement (Diercke et al., 2011). Hence, to our knowledge we are the first to report on the ephrin expression profile in TSPC. Among the 15 Eph receptors and the nine EFN ligands found in human, TSPC expressed predominantly four receptors and two ligands. Furthermore, upon tendon aging and degeneration, EphA4, EphB2, and EphB4 and EFNB1 ligand were significantly downregulated, while EFNB2 was upregulated, both on mRNA and protein levels. After initial screening of the downregulated ephrin candidates, we decided to focus in the main part of our study on rescue experiments with EphA4-Fc and EphB2-Fc proteins and to investigate their effect on the self-renewal, migration and actin dynamics of A-TSPC.

We started with self-renewal analyses. Previous literature on different ephrin members has suggested strong cell-specific effects; for example, EphA7/EFNA2 negatively regulates the cell proliferation of adult neural progenitor in the olfactory bulb (Holmberg et al., 2005), whereas EphB2-forward signaling stimulates intestine progenitor self-renewal (Genander et al., 2009). When we examined TSPC self-renewal by performing short-term cell proliferation and CFU assays, we found that EphA4-Fc, but not EphB2, can rescue the age-associated drop of proliferation as well as demonstrated a tendency to elevate A-TSPC clonogenicity, a finding which can be further explored and optimized in follow up studies. Our investigation of the downstream effectors revealed that both clustered proteins trigger activation of JNK kinase, however, EphA4-Fc also elevates the FAK phosphorylation, while EphB2 p38 phosphorylation. Hence, we speculate that this divergence in their signaling cascades might be contributing for the different effect on A-TSPC self-renewal; however, follow up studies will be necessary to investigate in great details the exact molecular mechanisms.

Next, we focused on investigating the effect of EphA4-Fc and EphB2-Fc on A-TSPC migration and actin turnover. We have described the age-related marked reduction in cell motility and actin dynamics of TSPC in (Kohler et al., 2013); here, we report for the first time that both EphA4-Fc or EphB2-Fc can significantly increase A-TSPC random migration and wound healing to rates similar to, and in the case of EphA4-Fc even better than, that of Y-TSPC. This novel data is in line with several studies claiming a positive effect of different ephrin family members on stem or progenitor cell migration (Arthur et al., 2011; Goichberg et al., 2011; Li and Johnson, 2013). Actin cytoskeletal dynamics are pivotal for cell migration (Rottner and Stradal, 2011), therefore by implementing latrunculin A experiments and F-actin staining we compared the cell shapes, F-actin content and the kinetic, and speed of actin filament turnover in Y-TSPC versus A-TSPC with or without EphA4-Fc and EphB2-Fc. Our results clearly demonstrated that activation of EphA4 or EphB2 reverse signaling in A-TSPC significantly improves their actin turnover to a mode similar to Y-TSPC. However, the A-TPSC shape and F-actin amount at the start point of the experiments were not affected by the addition of EphA4-Fc and EphB2-Fc, a finding that is different to other reports showing a regulatory function of certain ephrins on cell shape and spreading (Stokowski et al., 2007; Yamazaki et al., 2009; Arthur et al., 2011). It has been proposed that ephrins can affect cell migration by interfering with actin cytoskeleton dynamics via modulated GTPase activity (Marston et al., 2003; Buricchi et al., 2007; Takeuchi et al., 2015). A current limitation of our study is the lack of specification of EphA4 and EphB2 reverse partners in TSPC. Based on the expression analyses, we can speculate that the major ligands are EFNB1 or EFNB2, however, the identification of the exact EphA4 or EphB2-dependent reverse signaling pathway, and the possible involvement of GTPases, remain as challenges to be addressed in future research.

Taken together, our novel data suggests that decreased expression of the ephrin receptors EphA4 and EphB2 during tendon aging and degeneration limits the establishment of appropriate cell–cell interactions between TSPC and significantly diminished their proliferation, motility and actin turnover. We found that delivery of EphA4-Fc or EphB2-Fc to A-TSPC can successfully normalize several of the key aspects of their age-related phenotype and that in-between the two the EphA4-Fc is the more dominant. We could propose that dysregulation in EphA4-trigered bi-directional signaling may contribute to the inferior and delayed tendon healing common for aged individuals, which will be the focus for upcoming investigation. Hence, we believe that our pioneering study provides important and novel insight into the complex nature of tendon aging and degeneration.

## Author Contributions

CP and DD contributed to analysis and interpretation of data and design of the work, CP and JK contributed to acquisition of data; CP and DD were involved in drafting of the work and in the work revision; all authors were involved in the final approval of the manuscript and are agreeing to be accountable for all aspects of the work and ensuring that questions related to the accuracy or integrity of any part of the work are appropriately investigated and resolved.

## Conflict of Interest Statement

The authors declare that the research was conducted in the absence of any commercial or financial relationships that could be construed as a potential conflict of interest.

## Supplementary Material

FIGURE S1 **| Complete western blot image**. Aliquots of equal amounts of protein from three Y-TSPC and A-TSPC treated with EphA4-, EphB2-, EphB4-, and EFNB1-Fc proteins were loaded onto the same western blot membranes, which were first probed for the expression levels of phospho-FAK, ERK, AKT, Jnk, and p38. Next, the same membranes were stripped and probed for the corresponding total-FAK, ERK, AKT, Jnk, and p38. Comparable protein loading was validated with anti-GAPDH antibody. Complete western blot image corresponding to the phosphor-lanes in the inset B of **Figure 2**. The same procedure was performed for another two A-TSPC donors. Abbreviations: MW – positions of the molecular weight protein standard; 1–3, different Y-TSPC donors; A4, EphA4-Fc-; B2, EphB2-Fc-; B4, EphB4-Fc; B1, EFNB1-Fc-treated, and “-”, non-treated A-TSPC.
