# Supplementary figures and images for: Activation of EphA4 and EphB2 Reverse Signaling Restores the Age-Associated Reduction of Self-Renewal, Migration, and Actin Turnover in Human Tendon Stem/Progenitor Cells
Source: Front Aging Neurosci. 2016 Jan 6;7:246. doi: 10.3389/fnagi.2015.00246 (PMC4701947; doi:10.3389/fnagi.2015.00246)

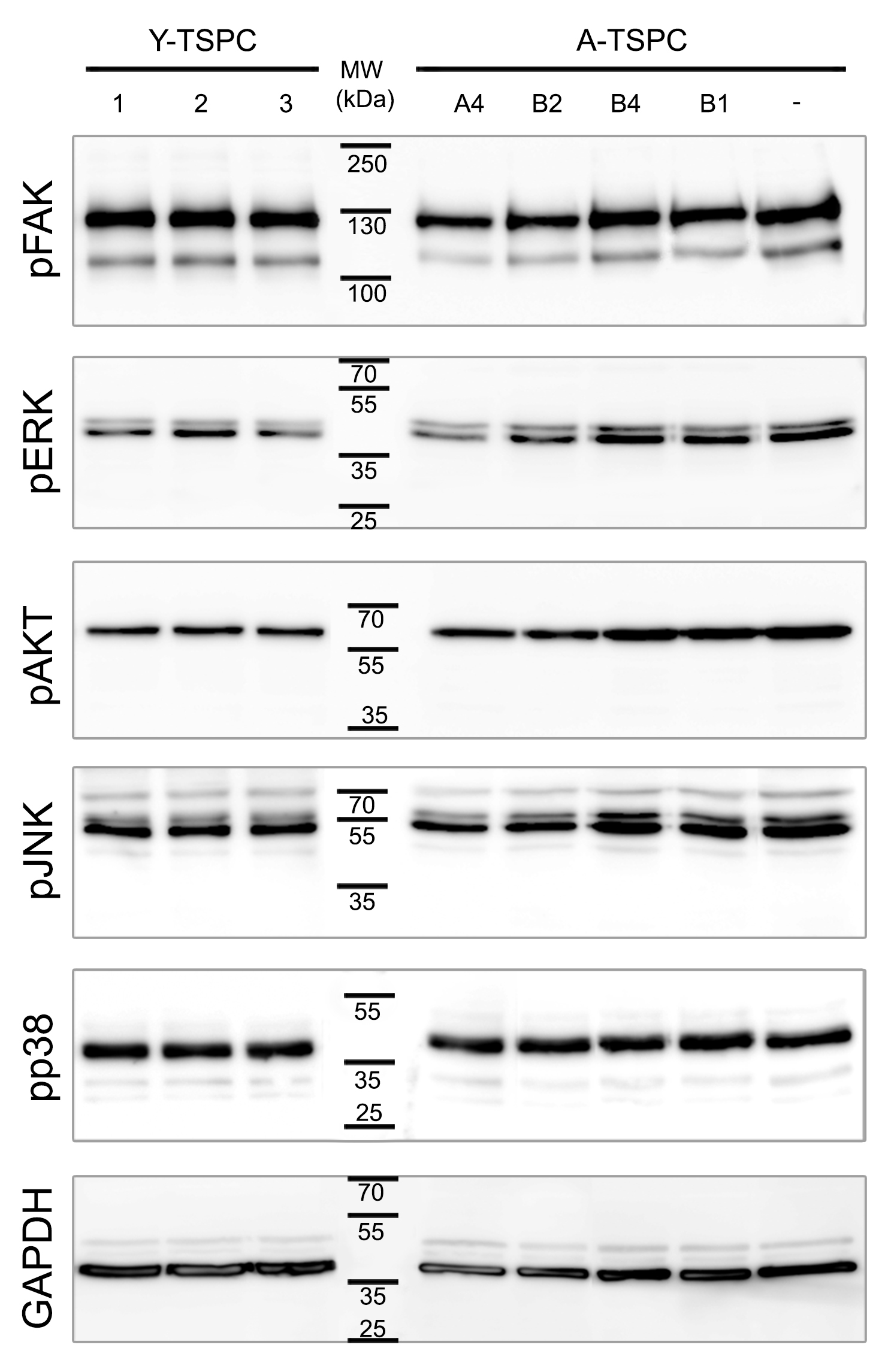

Supplement: FIGURE S1 — | Complete western blot image. Aliquots of equal amounts of protein from three Y-TSPC and A-TSPC treated with EphA4-, EphB2-, EphB4-, and EFNB1-Fc proteins were loaded onto the same western blot membranes, which were first probed for the expression levels of phospho-FAK, ERK, AKT, Jnk, and p38. Next, the same membranes were stripped and probed for the corresponding total-FAK, ERK, AKT, Jnk, and p38. Comparable protein loading was validated with anti-GAPDH antibody. Complete western blot image corresponding to the phosphor-lanes in the inset B of Figure 2. The same procedure was performed for another two A-TSPC donors. Abbreviations: MW – positions of the molecular weight protein standard; 1–3, different Y-TSPC donors; A4, EphA4-Fc-; B2, EphB2-Fc-; B4, EphB4-Fc; B1, EFNB1-Fc-treated, and “-”, non-treated A-TSPC. [file Image_1.JPEG]
